# Satellite DNA evolution in Tytonidae (Aves: Strigiformes): dynamic repeat landscapes despite conserved karyotypes

**DOI:** 10.1007/s10577-026-09817-2

**Published:** 2026-09-17

**Authors:** Felipe Lagreca Bitencourt, Gustavo Akira Toma, Luciano Cesar Pozzobon, Victor Cruz Cuervo, Ricardo Utsunomia, Maria Eduarda Gisloti-Ribeiro, Michel Octávio Fiorenço Capana, Raqueli Teresinha França, Marcelo de Bello Cioffi, Thales Renato Ochotorena de Freitas, Rafael Kretschmer

**Affiliations:** 1 Laboratório de Citogenética e Evolução, Departamento de Genética, Instituto de Biociências, Universidade Federal do Rio Grande do Sul, Porto Alegre, RS 91509-900 Brazil; 2 Laboratório de Citogenética Evolutiva, Departamento de Genética e Evolução, Universidade Federal de São Carlos, São Carlos, 13565-905 Brazil; 3 Faculdade de Ciências, Universidade Estadual Paulista, Bauru, SP 13506-900 Brazil; 4 Departamento de Clínicas Veterinária, Faculdade de Veterinária, Universidade Federal de Pelotas, Pelotas, RS Brazil

**Keywords:** Strigiformes, Karyotype evolution, Repetitive DNA, Heterochromatin, FISH mapping, Genome organization

## Abstract

The elevated chromosome numbers observed in Tytonidae relative to the putative ancestral avian karyotype suggest that lineage-specific chromosomal fissions may have played an important role in the evolutionary history of this family. Here, we provide the first cytogenetic characterization of the American barn owl (*Tyto furcata*) and performs a comparative repeatome analysis across members of the Tytonidae, including other two species, the Western barn owl (*Tyto alba*), and the Oriental bay owl (*Phodilus badius*). The karyotype of *T*. *furcata* showed a 2n = 92, closely resembling that previously described for *T*. *alba*, indicating a high degree of chromosomal conservation within Tytonidae. Although *T*. *furcata* and *T*. *alba* exhibit similar karyotypic organization, comparative repeatomic analyses revealed differences in their composition, including variation in satellite DNA (satDNA) repertoires and abundance. Eight satDNA families were identified in *T*. *furcata*, nine in *T*. *alba*, and 28 in *P*. *badius*, highlighting the dynamic evolution of repetitive sequences. Several satDNA families were shared between *T*. *furcata* and *T*. *alba*, whereas some appeared species-specific, supporting the library hypothesis of satDNA evolution. In *P*. *badius*, multiple satDNAs exhibited similarity to transposable elements, suggesting that mobile elements contributed to their diversification. Cytogenetic analyses demonstrated centromeric heterochromatin distribution in *T*. *furcata*, as well as a large heterochromatic W chromosome enriched in DNA repeats. The localization of satDNAs in centromeric regions and the apparent accumulation of repeats on the W chromosome reinforce the role of repetitive sequences in chromosome organization and sex chromosome differentiation. Together, these findings reveal repeatome diversification despite conserved macrochromosomal structure and provide new insights into genome evolution and chromosomal dynamics in birds.

**Supplementary Information:**

The online version contains supplementary material available at https://doi.org/10.1007/s10577-026-09817-2.

## Introduction

Birds exhibit one of the most conserved karyotypes among vertebrates, typically with a diploid number (2n) of approximately 80 and a clear distinction between macro- and microchromosomes (Griffin et al. 2007, 2025; Kretschmer et al. 2018a; O’Connor et al. 2024). This organization is considered ancestral for birds and reflects strong evolutionary constraints on chromosome structure. Nevertheless, some avian lineages deviate markedly from this pattern, displaying extensive chromosomal rearrangements and wide variation in 2n making them valuable systems for investigating genome evolution and speciation (Griffin et al. 2007, 2025; Kretschmer et al. 2018a; O’Connor et al. 2024).

Chromosomal rearrangements, including fissions, fusions, inversions, and translocations, are key drivers of genome evolution and may contribute to reproductive isolation by reshaping recombination landscapes and generating genomic incompatibilities (Rieseberg 2001; Faria and Navarro 2010). Although most birds exhibit karyotypic stability, groups such as Falconiformes, Psittaciformes, and Strigiformes show accelerated chromosomal evolution, suggesting lineage-specific influences on genome architecture (Kretschmer et al. 2018a; O’Connor et al. 2019; Griffin et al. 2025). Strigiformes (owls) are particularly notable in this context due to their pronounced karyotypic diversity (Bian et al. 1991; Renzoni and Vegni-Talluri 1966; Hassan 1998). The order comprises approximately 255 species distributed across Strigidae and Tytonidae (Gill et al. 2025). Within Tytonidae (barn owls), 2n range from 50 in *Tyto capensis* (Bian et al. 1991) to 92 chromosomes in *T*. *alba* (Renzoni and Vegni-Talluri 1966; Hassan 1998), although only a few species have been karyotyped to date (Degrandi et al. 2020). This wide variation indicates extensive chromosomal reorganization and highlights barn owls as a promising system for studying genome evolution.

The taxonomy of barn owls has long been debated, particularly within the *T*. *alba* complex (König et al. 2008; Aliabadian et al. 2016; Uva et al. 2018; Daugherty et al. 2026). Although traditionally treated as a single cosmopolitan species, recent integrative analyses support the recognition of three distinct lineages: *T. alba* in Europe, Africa, and parts of the Middle East; *T. furcata* throughout the Americas; and *T. javanica* across Southeast Asia and Oceania (Uva et al. 2018; Daugherty et al. 2026). Notably, earlier cytogenetic studies reporting 2n = 92 for *T*. *alba* were based on specimens from Italy and Egypt, which correspond to *T*. *alba* under this revised classification. This framework is essential for interpreting cytogenetic data, as independently evolving lineages may exhibit distinct patterns of chromosomal organization. However, while the karyotype of *T. alba* is relatively well characterized, those of *T. furcata* and *T. javanica* remain completely unknown, a critical gap in our understanding of karyotypic evolution across this species complex.

Repetitive DNA, including satellite DNAs (satDNAs), transposable elements (TEs), and ribosomal DNA arrays, constitutes a substantial fraction of eukaryotic genomes and plays central roles in chromosome structure and function (Ugarković 2005; Garrido-Ramos 2017; Ribas et al. 2026). SatDNAs are major components of constitutive heterochromatin, typically concentrated in centromeric and pericentromeric regions, where they contribute to kinetochore formation and chromosomal stability (Garrido-Ramos 2017). Satellite DNA evolves rapidly through mechanisms including unequal crossing-over and gene conversion, giving rise to lineage-specific repeat landscapes that may contribute to chromosomal rearrangements (Plohl et al. 2012). Likewise, ribosomal DNA (rDNA) arrays are highly dynamic genomic regions and have been associated with chromosome breakage and structural reorganization (Kobayashi 2011).

Here, we integrate cytogenetic analyses and genome sequencing to investigate the distribution and evolutionary dynamics of repetitive DNAs in *T*. *furcata*. We characterize the karyotype, map constitutive heterochromatin and ribosomal DNA clusters, and investigate repetitive DNA organization through repeatome and satellitome analyses, complemented by fluorescence in situ hybridization to localize satDNA families. In addition, we perform in silico analyses of the satellitomes of *T*. *alba* and *Phodilus badius*, establishing a comparative framework to explore the evolution of satDNA within Tytonidae. We further compare our findings with available cytogenetic data for *T*. *alba*, including populations from Egypt and Italy. By linking chromosomal architecture to satellitome variation, our study provides new insights into the mechanisms underlying karyotype diversification in Strigiformes and advances our understanding of genome evolution in birds.

## Material and methods

### Biological samples, chromosomal preparation, and C-banding

Six individuals of *T*. *furcata*, comprising two males and four females, were obtained in the municipality of Pelotas, Rio Grande do Sul, Brazil, from the Núcleo de Reabilitação da Fauna Silvestre at the Universidade Federal de Pelotas (31° 48′ 08″ S, 52° 25′ 34″ W). Sample collection and all technical procedures were carried out under authorization of the Brazilian Environmental Agency ICMBio/SISBIO (license number 81564-1). All procedures complied with ethical guidelines and were approved by the Animal Ethics Committee of the Universidade Federal de Pelotas (protocol 113/2023). Primary fibroblast cell cultures were established from feather-derived samples (Cioffi et al. 2026), using Dulbecco’s Modified Eagle’s Medium (DMEM) supplemented with 20% fetal bovine serum and 1% penicillin–streptomycin, and maintained at 37 °C under standard culture conditions. Metaphase chromosomes were obtained according to standard cytogenetic protocols, including colcemid treatment (1 h), hypotonic treatment in 0.075 M KCl (10 min), and fixation in methanol:acetic acid (3:1), also following Cioffi et al (2026). After fixation, at least 20 metaphase spreads stained with 5% Giemsa in 0.07 M phosphate buffer (pH 6.8) were analyzed for karyotype characterization. Chromosomes were classified according to Guerra (1986). C-banding was performed following the protocol described by Sumner (1972) for the detection of constitutive heterochromatin, with modifications as proposed by Lui et al. (2012).

### DNA extraction and genomic sequencing

Genomic DNA (gDNA) was extracted from feather samples of one male and one female using the PureLink™ Genomic DNA Mini Kit (Invitrogen, Carlsbad, CA, USA), following the manufacturer’s instructions and the method of Sambrook and Russell (2001). Whole-genome sequencing was performed on the BGISEQ-500 platform (BGI Shenzhen Corporation, Shenzhen, China) using gDNA from both individuals of *T*. *furcata*. Each library generated approximately 3 Gb of data, yielding 150 bp paired-end reads. The resulting sequence data have been deposited in the Sequence Read Archive (SRA) under the accession numbers SRR38417748 (female individual) and SRR38417747 (male individual).

To conduct a comparative genomic analysis within Tytonidae, additional short-read datasets were retrieved from the SRA database for two species of barn owls: *T*. *alba* (female: SRR30978371; male: SRR30978505) and *P*. *badius* (sex unknown: SRR28397836). These datasets represent the only short-read whole-genome sequencing data currently available for the family.

### Characterization of repeatomes in the barn owls

For a comprehensive comparison and characterization of the barn owl repeatome, we analyzed sequencing data from *T*. *furcata* together with previously available read libraries for *T*. *alba* and *P*. *badius*. We performed multiple iterative rounds of satDNA identification and annotation using a graph-based clustering algorithm implemented in the RepeatExplorer and Tandem Repeat Analyzer (TAREAN) pipelines, accessible via the Galaxy server. Prior to analysis, all sequencing reads were quality-filtered (Q < 20) and trimmed using Trimmomatic v.3.0 (Bolger et al. 2014) with the following parameters LEADING:3, TRAILING:3, SLIDINGWINDOW:4:20, MINLEN:100, and CROP. A subset of 2 × 500,000 paired‑end reads from the female genomes of *T*. *furcata* and *T. alba* and from the genome of *P. badius* was used for TAREAN analysis with default parameters, a minimum cluster size of 0.001%, and the run queue set to extra‑long. Annotated sequences with circular topology graphs were considered putative satDNAs and selected for downstream analysis. Recovered satDNAs were subsequently purged from their corresponding trimmed reads using Deconseq v0.4.3 (Schmieder and Edwards 2011). To detect remaining repeats, a fresh subsample of 2 × 500,000 paired reads was prepared from each sequencing library and cycled through alternating rounds of TAREAN and Deconseq filtering. This process was repeated until no new satDNA sequences emerged. Finally, we screened each satellitome dataset for non-satDNA repetitive DNA (e.g., multigene families, TEs). All satDNA catalogs were deposited on the Genbank with accession numbers: PZ387877 to PZ387921.

TE characterization was carried out with DNApipeTE v1.4 (Goubert et al. 2015), a tool optimized for repeat detection from low-coverage short-read data. Single-end forward reads from each trimmed library served as input, and default parameters were applied alongside a 0.2 × genome coverage setting. Based on previously assembled genome sizes (1.2 Gb for *T. alba* and *T. furcata*, GCA_018691265.1 and 1.5 Gb for *P. badius*, GCA_040207595.1), we estimated the relative proportion of each repetitive DNA class in the three species genomes.

### Estimating the abundance, divergence, and homology of satellite DNAs

The relative abundance of each satDNA was obtained by dividing the number of satDNA‑derived nucleotides by the total genomic nucleotides surveyed, based on a 2 × 5,000,000 paired‑end read sample per genome. Alignments of recovered satDNAs to their respective genomes were carried out with the "cross‑match" tool within RepeatMasker v.4.16 (Smit et al. 2020), in combination with a custom Python script (https://github.com/fjruizruano/ngs-protocols/blob/master/repeat_masker_run_big.py). Genetic distances were then estimated under the Kimura‑2‑parameter (K2P) model (Kimura 1980) using the calcDivergenceFromAlign.py script from RepeatMasker (Smit et al. 2020).

To identify sex-biased satDNAs in *T. alba* and T*. furcata,* we calculated the female-to-male abundance ratio (F/M) for each satDNA sequence using RepeatMasker outputs. SatDNAs with an F/M ratio greater than 1 were considered female-biased.

Satellite DNAs were ranked by their genomic abundance (from highest to lowest) and named according to the criteria proposed by Ruiz-Ruano et al. (2016). Thus, *T. alba* and *P. badius* satDNAs were named TalSatDNAs and PbaSatDNAs, respectively. To evaluate cross‑species conservation of satDNA sequences and identify TE insertions and homology groups for each satDNA, similarity searches were performed using a custom Python script rm_homology.py (https://github.com/fjruizruano/satminer/blob/master/rm_homology.py) (Ruiz-Ruano et al. 2016), followed by manual sequence alignments and BLASTn searches (Altschul et al. 1990) against the NCBI/GenBank and RepBase GIRI/CENSOR databases (Vertebrate and *Gallus gallus* sequence source mode). All satDNAs were then classified as variants (identity > 95%), families (80–95%), and superfamilies (SF) (50–80%) (Ruiz-Ruano et al. 2016).

### Using PacBio data to support Illumina reads

To validate satDNA abundance estimates obtained from Illumina short reads, we analyzed a publicly available PacBio HiFi dataset from *T*. *alba* (SRR37817609), comprising 1,639,724 reads and 37.34 Gb of sequence (~ 31 × coverage assuming a 1.2 Gb genome). Read quality was assessed with NanoPlot software (De Coster et al. 2018), and residual adapter sequences were evaluated using the PacBio HiFi adapter filter (Sim et al. 2022) available through Galaxy platform. The filtered reads were randomly subsampled into three independent datasets of 1.5 Gb each using seqtk (Li 2012; https://github.com/lh3/seqtk) with seeds 100, 200 and 300, matching the amount of sequence analyzed in the Illumina dataset (2 × 5,000,000 paired-end reads of 150 bp; ~ 1.5 Gb).

SatDNA abundance was estimated with RepeatMasker v4.1.6 (Smit et al. 2020) using a custom library containing dimerized consensus sequences of the nine *T. alba* satDNA families and the RMBlast search engine (Morgulis et al. 2006). Hits were assigned to satDNA families, and overlapping alignments were merged using the IRanges R package (Lawrence et al. 2013), ensuring that overlapping annotations within the same read were counted only once. Abundance was calculated as the proportion of nucleotides assigned to each satellite family relative to the total number of nucleotides analyzed (1.5 Gb), following the same procedure used for the Illumina data. Estimates were obtained for each subsample independently, and mean values and standard deviations were calculated across the three replicates.

### Using the scaffold-level PacBio assembly to assess satDNA representation in the genome assembly

To investigate the representation of TalSatDNAs in the *T. alba* scaffold-level genome assembly (GCF_018691265.1), we performed a standard RepeatMasker (Smit et al. 2020) analysis using the multimerized consensus library of the nine *T. alba* satDNA families. For each annotation, we recorded the scaffold, genomic coordinates, family identity, and alignment length, which were used to estimate satDNA representation in the assembly.

### Satellite DNA and 18S rDNA gene amplification and chromosomal mapping

We selected five of the eight satDNA families identified in *T. furcata* for primer design based on their consensus monomer sequences (Table S1). The three extant satDNA families (named TfuSat02-28, TfuSat03-28, and TfuSat04-21) were synthesized and labeled with biotin at the 5′ end by Exxtend (Paulínia, Brazil) due to their short monomer lengths (28, 28, and 21 bp, respectively).

PCR amplification of TfuSatDNAs was performed under the following conditions: an initial denaturation at 95 °C for 5 min; 40 cycles of denaturation at 95 °C for 40 s, annealing at 51–57 °C for 35 s, and extension at 72 °C for 1 min; followed by a final extension at 72 °C for 7 min (Table S2). Amplification quality and product integrity were verified by electrophoresis on a 2% agarose gel. Additionally, genomic DNA from the emu (*Dromaius novaehollandiae*) was used as a template for PCR amplification of the 18S rDNA gene, following the protocol described by Kretschmer et al. (2022).

All TfuSatDNAs were labeled with biotin using the BioNick™ DNA Labeling System (Invitrogen, Carlsbad, CA, USA), following the manufacturer’s instructions. Fluorescence in situ Hybridization (FISH) experiments were carried out by standard protocol described by Kretschmer et al. (2022). Biotin-labeled probes were identified with the help of Streptavidin-Cy3 dye (Invitrogen, Thermo Fisher Scientific, Waltham, MA, USA), while the 4’,6’-diamidino-2-phenylindole (DAPI; Sigma-Aldrich, St. Louis, MO, USA) solution was used to counterstain the chromosomes. Images of at least 15 metaphase spreads were captured and analyzed using an AxioCam MRm camera and ZEN 2 software (Blue edition) (ZEISS Inc., Carl Zeiss, Heidelberg, Germany) mounted on a Zeiss Axiophot fluorescence microscope (ZEISS Inc.). Final image processing and figure assembly were performed using CorelDRAW v.23.1.0.389.

## Results

### Karyotype characterization

**Fig. 1:**
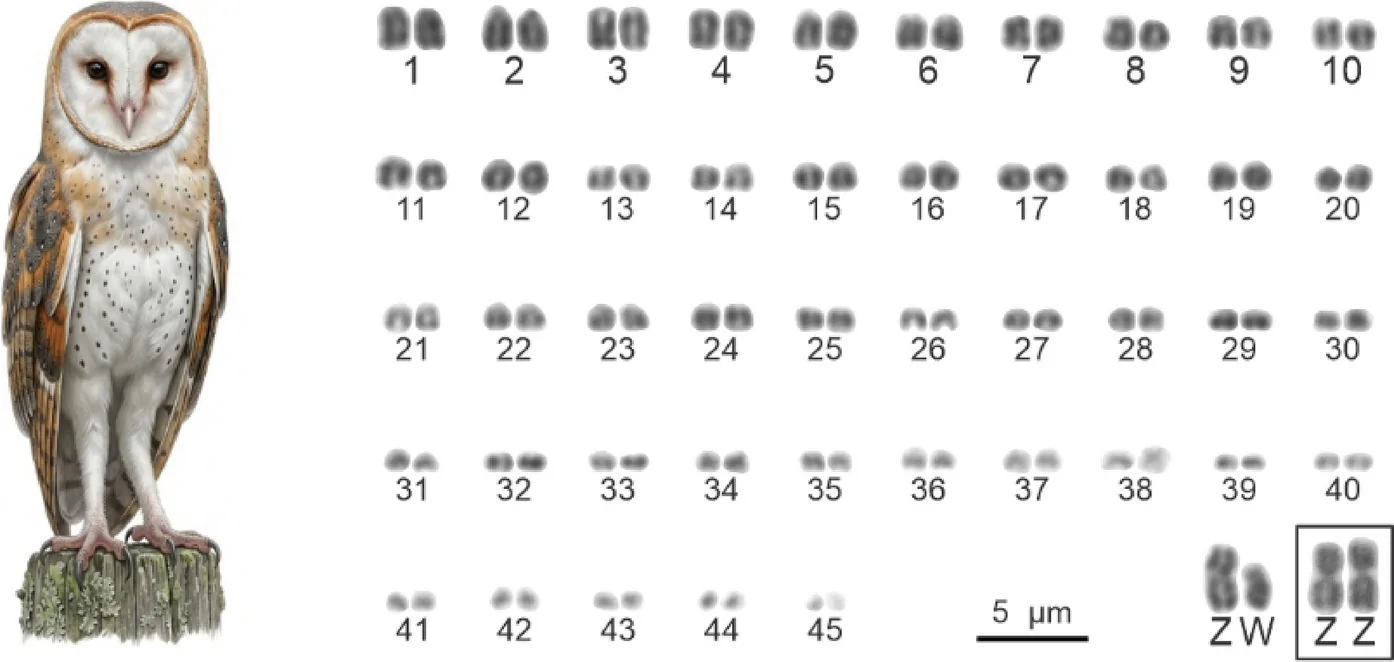
Giemsa-stained complete karyotype of a female *T. furcata*. For comparison, the male sex chromosome constitution (ZZ) is highlighted. Illustration of *T. furcata* generated with Gemini (Google) using the Nano Banana Pro model

Karyotype analysis using Giemsa-stained chromosome spreads revealed that the diploid number of *T. furcata* is 2n = 92 (Fig. 1). The chromosomes exhibit a gradual decrease in length, with all autosomes showing acrocentric morphology. The Z sex chromosome is the largest element in the karyotype, significantly exceeding the size of the 1 st pair, and is the only chromosome displaying a clearly metacentric morphology, making it readily distinguishable. The submetacentric W chromosome appears to be similar in size to the second chromosome pair (Fig. 1, boxed).

**Fig. 2:**
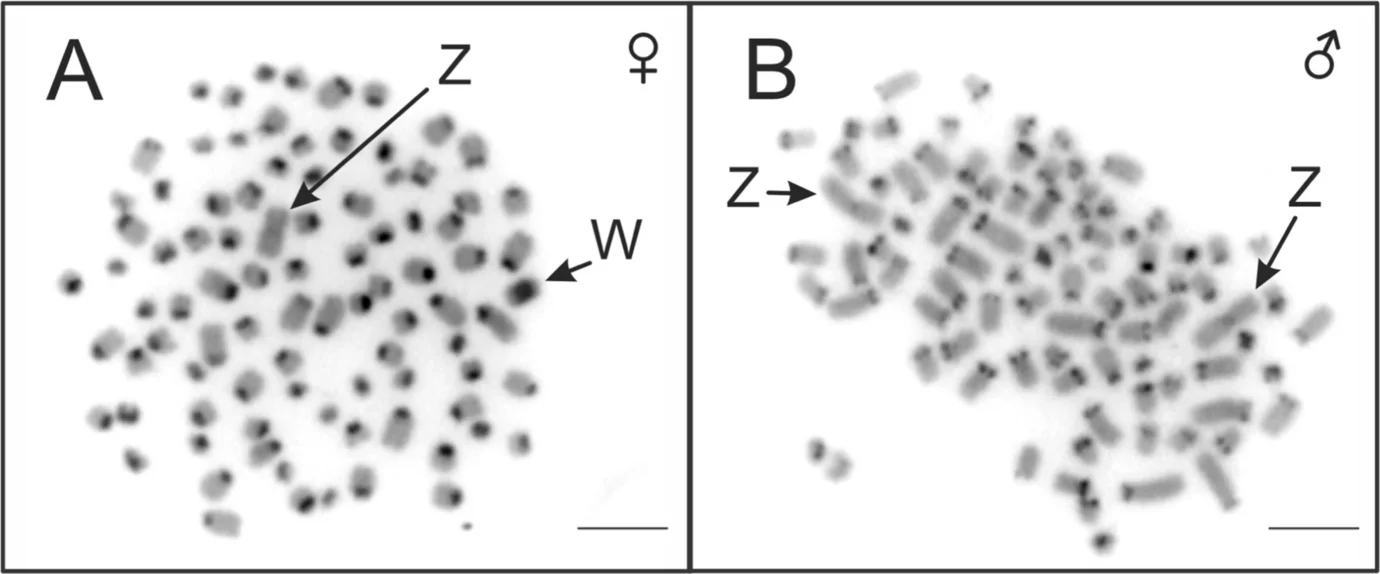
C-banding pattern in a female (**A**) and a male (**B**) of *T. furcata*. Sex chromosomes are indicated by arrows. Scale bar = 5 μm

Constitutive heterochromatin was predominantly localized in the centromeric regions of all macro- and microchromosomes, as well as on the W chromosome. A faint C-positive heterochromatic block was observed in the centromere of the Z sex chromosome (Fig. 2).

### Profiling and comparison of barn owls repeatomes

The genomes examined exhibited comparable levels of repetitive DNA, with total repeat content ranging from 11.14% to 12.80% (Table 1). LINE retrotransposons represented the most abundant class across all samples, accounting for 4.81% to 4.97% of the genomes. Within this category, elements belonging to the L2/CR1/Rex superfamily were the most prevalent. LTR elements also contributed substantially to the repetitive fraction, with values ranging from 4.54% to 4.98%, predominantly represented by retroviral elements. In contrast, SINEs, DNA transposons, and small RNA repeats comprised only minor proportions of the genomes, each accounting for less than 1% of the total genomic content. Overall, these findings indicate a conserved pattern of repetitive element composition among the analyzed species, characterized by the predominance of retrotransposons and the relatively low abundance of other repetitive DNA classes. Nevertheless, this apparent conservation did not extend to satDNAs, whose composition varied considerably among species, both in terms of the number of satellite DNA families identified and their genomic representation (Table S3, S4, and S5).

**Table 1:** Composition and relative abundance of repetitive elements in the analyzed genomes

	*Phodilus badius*	*Tyto alba (female)*	*Tyto alba (male)*	*Tyto furcata (female)*	*Tyto furcata (male)*
Retroelements	9.78	9.85	9.58	10.08	10.76
SINEs:	0.13	0.06	0.07	0.08	0.06
Penelope	0.00	0.01	0.01	0.00	0.00
LINEs:	4.82	4.81	4.97	5.35	5.81
L2/CR1/Rex	4.63	4.74	4.89	5.25	5.72
R2/R4/NeSL	0.00	0.01	0.02	0.07	0.04
RTE/Bov-B	0.00	0.02	0.03	0.01	0.01
L1/CIN4	0.18	0.02	0.02	0.02	0.02
LTR Elements:	4.84	4.98	4.54	4.65	4.89
Gypsy/DIRS1	0.02	0.01	0.00	0.01	0.01
Retroviral	4.79	4.96	4.50	4.61	4.86
DNA transposons	0.11	0.14	0.14	0.16	0.11
Hobo-Activator	0.00	0.04	0.03	0.04	0.03
Tc1-IS630-Pogo	0.00	0.01	0.01	0.01	0.01
Tourist/Harbinger	0.01	0.02	0.02	0.01	0.01
Rolling-circles	0.01	0.00	0.00	0.00	0.00
Unclassified repeats	0.10	0.09	0.07	0.05	0.06
Interspersed repeats	9.99	10.08	9.80	10.28	10.93
Small RNA	0.32	0.16	0.14	0.09	0.12
Satellites	0.41	0.17	0.08	0.08	0.09
Simple repeats	1.08	1.55	1.22	0.55	0.54
Low complexity	0.31	0.85	0.49	0.14	0.12
Total repetitive DNA	12.11	12.80	11.71	11.14	11.78

### Satellitome characterization in barn owls

In *T. furcata*, eight satDNA families were identified (Table S3). These families exhibited repeat unit lengths (RULs) ranging from 21 to 267 bp, with an average of 90 bp. Only two families (TfuSat1-162 and TfuSat8-267) were characterized by repeat units larger than 100 bp, whereas the remaining families consisted of short monomers (< 100 bp), with TfuSat4-21 being the smallest monomer size (Table S3). Regarding adenine and thymine content (A + T), four TfuSatDNAs (TfuSat01-162, TfuSat02-28, TfuSat06-80, and TfuSat08-267) exhibited A + T contents exceeding 50%. In contrast, the remaining families showed a considerably lower average of 36.3% (Table S3). The female and male individuals exhibited virtually identical genomic proportions of satDNAs, accounting for 3.99% and 4.08% of their genomes, respectively. Additionally, TfuSat02-28, TfuSat04-21, TfuSat05-74, TfuSat06-80, and TfuSat07-66 were relatively more abundant in females, as determined by the female-to-male abundance ratio (F/M) (Table S3).

Nine satDNA families were identified in the *T. alba* satellitome, with monomer lengths ranging from 21 to 1,306 bp (Table S4). Only three TalSatDNA families exhibited an A + T content higher than 50%, whereas the remaining families showed a mean of 30.1% (Table S4). The genomic proportion of satDNAs was higher in females (6.18%) than in males (5.70%), with TalSat2-162 and TalSat6-66 being more abundant in the female genome (Table S4).

The characterization of the *P. badius* satellitome revealed 28 satDNA families, with monomer lengths (RULs) ranging from 24 to 1,308 bp (Table S5). PbaSat17-24 exhibited the shortest monomer length, along with nine other satellite DNA families with monomers shorter than 100 bp. The A + T content exceeded 50% in 12 PbaSatDNA families, whereas the remaining families showed a mean A + T content of 42% (Table S5).

Comparative analysis of the satellitomes of *T*. *alba* and *T*. *furcata* revealed the presence of five shared satDNA families showing 100% sequence similarity between species (Table S6). These conserved families included TalSat03-21/TfuSat04-21, TalSat02-162/TfuSat01-162, TalSat04-28/TfuSat03-28, TalSat05-28/TfuSat02-28, and TalSat06-66/TfuSat07-66. Except for TalSat03-21/TfuSat04-21, which belongs to the SF-5 superfamily (TalSat01-42, TalSat03-21, TfuSat04-21), the remaining shared satDNAs were not assigned to any previously defined SF. In contrast, the other satDNA families identified in each species appeared to be species-specific, including three exclusive families in *T*. *alba* and three in *T*. *furcata*. *P*. *badius*, however, exhibited a substantially larger number of apparently species-specific satDNA families. Consistent with this observation, K2P landscapes (Figures S1–S3) revealed uniformly low divergence values for *P. badius* satellitome, suggesting either a recent evolutionary origin or strong sequence homogenization of satDNA families. Meanwhile, the satellitomes of *T. alba* and *T. furcata* showed high K2P values (K2P > 15) for three satDNAs (TalSat01-42, TalSat03-21, and TfuSat01-21) indicative of substantial mutation accumulation, a feature of either ancient or rapidly evolving satDNAs (Figures S1–S3).

Similarity searches against DNA databases (RepBase GIRI/CENSOR and GenBank) revealed that several satDNA families shared sequence similarity with TEs and other repetitive DNA sequences (Table S7 and S8). PbaSat01-182 showed similarity to the centromeric tandem repeat (CENSTRIG) of the Blakiston’s fish owl (*Ketupa blakistoni*) (Table S8), while PbaSat03-729 was found highly similar to a W chromosome repeat sequence in three Strigidae owls (Table S7). Additionally, four satDNA families matched predicted mRNAs, including the *CCR9* gene, suggesting either functional relevance or a gene ancestry (Table S7). The remaining satDNAs exhibited match predominantly with interspersed repeats. In particular, PbaSat02-1308 displayed high similarity to the non-LTR retrotransposon CR1-J2 Pass, representing an 87% identity match.

### In situ chromosome distribution of SatDNAs and 18S rDNA gene

**Fig. 3:**
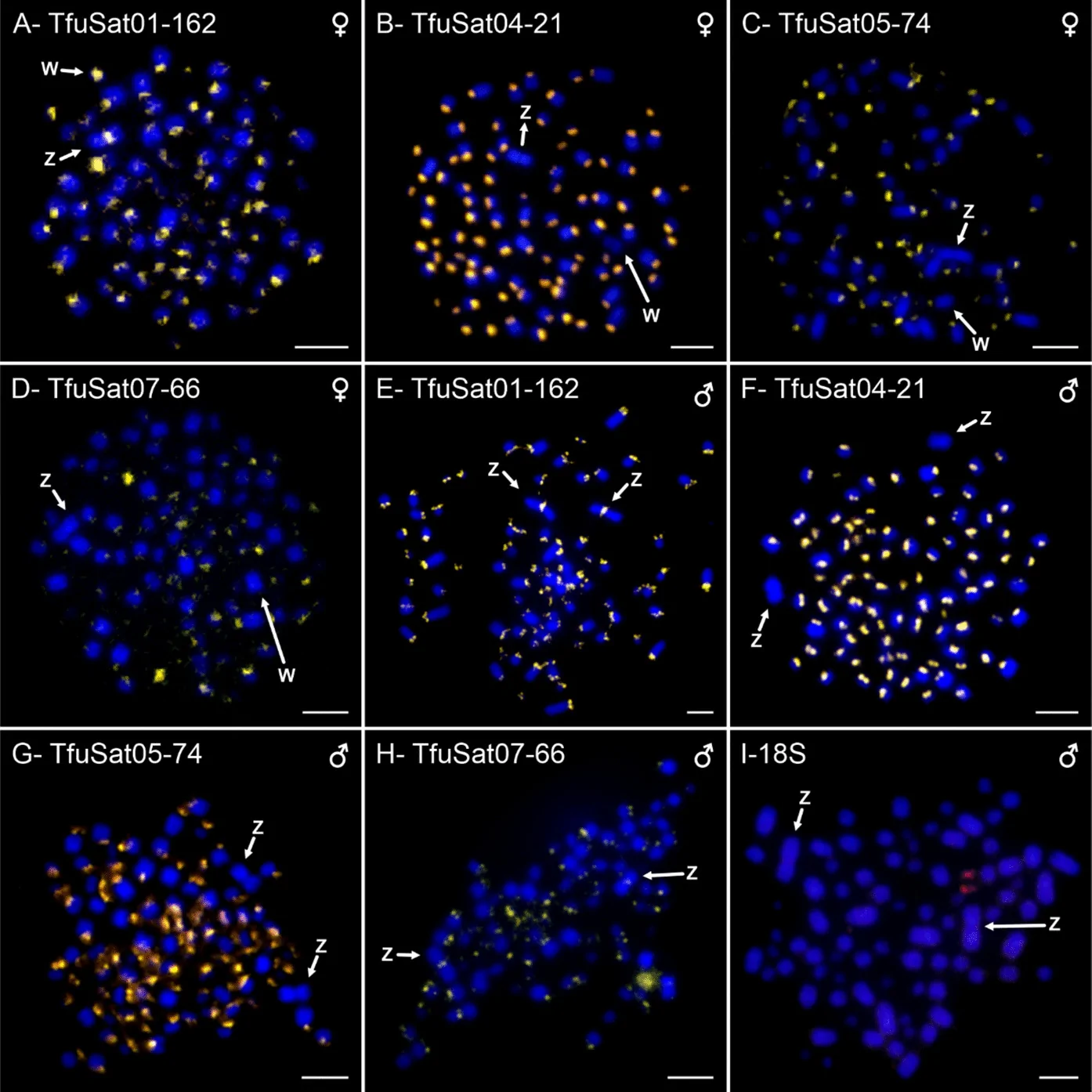
Metaphase chromosome spreads of *T. furcata* after fluorescence in situ hybridization (FISH) using TfuSatDNA probes (**A**–**H**) and hybridization signal of 18S rDNA probe (**I**). The chromosome probe and the individual sex used in each experiment is indicated in the upper corner of the respective panel. The sex chromosomes (Z and W) were identified based on their morphology and are highlighted by arrows in each panel. Scale bar = 5 μm

In situ hybridization experiments revealed that TfuSat01-162, TfuSat04-21, TfuSat05-74, and TfuSat07-66 produced clear hybridization signals on both female and male chromosomes of *T. furcata* (Fig. 3). Although TfuSat02-28 and TfuSat03-28 probes were successfully synthesized and biotin-labeled, no detectable FISH signals were observed (not shown). The remaining two satDNA (TfuSat06-80 and TfuSat08-267) could not be amplified and were therefore excluded from chromosomal mapping analyses.

TfuSat01-162 was prominently mapped to the centromeric/pericentromeric regions of all autosomes and also in both ZW sex chromosomes (Fig. 3A, E). TfuSat04-21 and TfuSat05-74 displayed similar hybridization patterns, predominantly localizing to the centromeres of macro- and microchromosomes; however, neither probe produced signals on the sex chromosomes (Fig. 3B, C, F, G). Likewise, TfuSat07-66 did not hybridize to the sex chromosomes but showed dispersed centromeric signals in at least five pairs of macrochromosomes and in several microchromosomes, with a particularly strong signal observed in one microchromosome pair (Fig. 3D, H). Additionally, FISH analysis identified the 18S rDNA cluster in one pair of microchromosomes (Fig. 3I).

Although TfuSat04-21 showed a relatively low genomic abundance estimate (~ 0.044%) (Table S3), it produced strong FISH signals (Fig. 3B and F). This apparent discrepancy highlights that FISH signal intensity is not necessarily proportional to the overall genomic abundance of a satellite DNA family. The chromosomal organization and local clustering of satellite arrays may influence signal intensity. Therefore, the abundance estimates obtained in silico should be interpreted as approximations, and additional independent quantitative approaches could provide further validation (see below).

## Independent validation of satDNA abundance using PacBio HiFi sequencing

To further validate satDNA abundance estimates and to assess the reliability of assembly-based quantification, we compared the nine TalSatDNA families across three independent sources: Illumina short reads, PacBio HiFi long reads, and the PacBio-based scaffold-level genome assembly (Table S9). Because the PacBio reads and the genome assembly derive from the same male individual (SRR37817609 and GCF_018691265.1, respectively), this pairing provides a controlled, within-individual test of assembly-based quantification, isolating the effect of genome assembly from biological variation.

Total satDNA content estimated directly from PacBio HiFi reads (13.53%) was roughly 520-fold higher than the value obtained from the corresponding genome assembly (0.026%). This discrepancy was consistent across families, with assembly-based estimates 1.8 × to more than 7,000-fold lower than the read-based estimate for the same individual (Table S9). The reduction was most pronounced for the two most abundant, shortest-monomer families, TalSat1-42 (42 bp) and TalSat3-21 (21 bp). This pattern is consistent with the collapse of highly identical tandem repeats during long-read genome assembly, a well-documented limitation of overlap-based assemblers when repeat units are shorter than, or comparable to, sequencing read length.

Comparison between Illumina short reads (male) and PacBio HiFi reads (male, same species but different individuals) showed good agreement in order of magnitude for five of the nine families (TalSat2-162, TalSat6-66, TalSat7-1316, TalSat8-145, and TalSat9-262; 0.26–4.55-fold difference). Four families showed larger differences: TalSat1-42 and TalSat3-21 were 2.8- and 5.2-fold more abundant in the PacBio individual, while TalSat4-28 and TalSat5-28 were nearly absent from the PacBio dataset relative to Illumina (approximately 500- and 1,250-fold lower, respectively).

## Discussion

### Repeatome and karyotype evolution in the barn owls

Here, we present the first karyotype description of the American barn owl *Tyto furcata*, together with the repeatome characterization of *T*. *furcata*, *T*. *alba*, and *P*. *badius*. The karyotype of *T*. *furcata* comprises a 2n of 92 chromosomes, closely matching the pattern previously reported for *T*. *alba* (Renzoni and Vegni-Talluri 1966; Belterman and De Boer 1984; Hassan 1998). This correspondence indicates that both taxa share similar chromosome numbers and overall karyotypic morphology, suggesting a high degree of chromosomal conservation despite their geographic separation and evolutionary divergence (approximately 5.0 million years ago) (Kumar et al. 2022). In addition, the localization of the 18S rDNA cluster on a pair of microchromosomes agrees with the pattern commonly observed in birds, in which ribosomal gene clusters are frequently associated with these elements (Nishida-Umehara et al. 2007). The maintenance of rDNA loci on microchromosomes may reflect selective pressures preserving their genomic organization.

From an evolutionary perspective, this similarity may reflect strong constraints acting on chromosomal architecture, possibly associated with the preservation of gene order (synteny) and regulatory interactions (Ellegren 2010). Such stability could indicate that at least large-scale chromosomal rearrangements are either selectively disadvantageous or have not been a major driver of divergence in these lineages. Alternatively, the shared karyotype may point to a relatively recent common ancestry, with insufficient time for the accumulation of detectable significant interchromosomal changes (Ellegren 2010; Poignet et al. 2021; Toma et al. 2023). At the same time, the 2n of 92 chromosomes in *T*. *furcata* and *T*. *alba* suggests extensive chromosomal reorganization relative to the putative ancestral avian karyotype, which is estimated to have approximately 80 chromosomes (Griffin et al. 2007; Griffin and Burt 2014; Kretschmer et al. 2018a; O’Connor et al. 2024). A similar pattern is also evident in *P*. *badius*, whose karyotype has been described as 2n = 90 (Belterman and De Boer 1984). In contrast, most Strigiform species exhibit a relatively conserved 2n ranging from 76 to 80 chromosomes (Degrandi et al. 2020). Therefore, the elevated 2n observed in Tytonidae likely originated through lineage-specific interchromosomal rearrangements, particularly macrochromosomes fissions, as reflected in the reduced number of biarmed chromosomes (Fig. 2), a feature that may characterize this family.

Despite the apparent interspecific karyotypic similarity between *T*. *furcata* and *T*. *alba*, and a relatively similar number of satDNA families some genomic differences can be observed, particularly in the composition and abundance of repetitive DNA sequences. For instance, three apparently exclusive satDNAs were identified in each species: *T. furcata* and *T. alba*. These results underscore the dynamic evolution of repetitive sequences and further support the recognition of these taxa as distinct species. Additionally, our findings demonstrate that variation in repeat content can accumulate even in the absence of major macrostructural chromosomal changes, corroborating previous studies showing that repetitive elements evolve rapidly (Henikoff 2001; Cara and Levine 2021).

The observed sequence similarities between satDNAs and TEs reinforce the growing evidence that repetitive DNA components are evolutionarily interconnected (Satović et al. 2016; Zattera and Bruschi 2022). In *P*. *badius*, two satDNA families exhibited similarity to interspersed repeats, suggesting that TEs may have contributed to their origin, amplification, and diversification. In contrast, no such clear association was detected in *T*. *furcata* or *T. alba*. This pattern suggests that TEs may have played a more prominent role in shaping the satellitome of *P*. *badius*, potentially contributing to the higher diversity of satDNA families identified in this species (28 families).

The identification of five satDNA families with identical sequences (100% identity matches) in *T*. *alba* and *T*. *furcata* indicates the conservation of part of the satellitome between these closely related species, likely reflecting shared ancestry and the maintenance of ancestral repetitive sequences during diversification. At the same time, the presence of species-specific satDNA families in both taxa suggests that differential amplification, sequence divergence, or loss of repetitive elements have occurred independently after speciation. This pattern was even more pronounced in *P*. *badius*, which exhibited a markedly larger number of apparently exclusive satDNA families (Table S5), indicating a more dynamic evolutionary trajectory of repetitive DNA in this lineage. Nevertheless, the apparent absence of shared satDNAs among species should be interpreted cautiously, since some sequences classified here as species-specific may still be present in other genomes at very low copy numbers and therefore remain undetected by the analytical in silico approaches employed. Such a scenario is consistent with the library hypothesis (Fry and Salser 1977), in which related species retain a common repertoire of satellite sequences that may undergo differential amplification or contraction over evolutionary time.

### Sex chromosome evolution on *Tyto furcata*

As previously observed in Strigidae (Yamada et al. 2004), constitutive heterochromatin in *T*. *furcata* is mainly associated with centromeric regions. In addition, a prominent C-positive heterochromatin block was detected on the W chromosome. This pattern is consistent with the well-established evolutionary differentiation of avian sex chromosomes, in which reduced recombination and sequence degeneration contribute to the accumulation of repetitive DNA and heterochromatin on the W chromosome (Schartl et al. 2016; Kretschmer et al. 2018b; Peona et al. 2021; Setti et al. 2024).

Both Z and W chromosomes of *T*. *furcata* are metacentric and substantially larger than the largest autosomal pair (Fig. 1), differing from the more typical avian pattern in which the Z chromosome is comparable in size to the largest macrochromosomes and the W is usually smaller and largely heterochromatic (Kretschmer et al. 2018a; Zhou et al. 2014). The prominent heterochromatic block on the W chromosome are consistent with an enrichment of repetitive sequences on this female-specific chromosome.

Finally, the absence of detectable FISH signals for some satellite families identified in genomic datasets may reflect a dispersed genomic distribution or relatively low copy numbers below the detection threshold of cytogenetic techniques. These discrepancies highlights the complementary resolution of genomic and chromosome-based analyses for investigating repetitive DNA (Ruiz-Ruano et al. 2016).

The comparison between read-based and assembly-based abundance estimates from the same individual demonstrates that the PacBio-based genome assembly substantially underrepresents TalSatDNA content, by roughly three to four orders of magnitude for the most abundant families. This result is consistent with the well-known tendency of long-read assemblers to collapse near-identical tandem repeat arrays into fewer copies than are truly present in the genome (Sproul et al. 2023; Nurk et al. 2022).

The residual differences observed between Illumina and PacBio HiFi read-based estimates for four families (TalSat1-42, TalSat3-21, TalSat4-28, and TalSat5-28) could not be explained by consensus library length or by a sex-specific effect, and most plausibly reflect genuine inter-individual variation in satDNA copy number, a phenomenon widely reported in satellite DNA arrays, which can expand or contract rapidly through unequal crossing-over and replication slippage even among individuals of the same population. We note this as a limitation of using single-individual sequencing data (whether short- or long-read) to characterize species-wide satDNA abundance, and we highlight it explicitly rather than treating either dataset as ground truth.

### Evolution of centromeric repeats

The genomic characterization of satellite DNAs further emphasizes the importance of repetitive sequences in shaping the chromosomal landscape of *T*. *furcata*. Satellite DNAs are major components of heterochromatin and frequently occupy centromeric or pericentromeric regions, where they contribute to chromosome stability and kinetochore function (Plohl et al. 2012; Garrido-Ramos 2017). The predominance of short repeat units (< 100 bp) among the identified satDNA families is consistent with patterns reported in several avian genomes, where centromeric satellites often consist of relatively short monomers that undergo rapid turnover (Kapusta and Suh 2017). Such structural features may facilitate dynamic amplification through mechanisms such as unequal crossing-over or replication slippage, promoting the rapid evolution of repetitive landscapes.

The centromeric localization of several satDNA families suggests that these sequences may contribute to the structural organization of centromeres in *T. furcata*. In particular, the widespread distribution of TfuSat01-162 across the centromeres of autosomes and sex chromosomes indicates that this repeat family may represent a major centromeric component in this species. Similar patterns have been reported in other vertebrates, where specific satDNA families become associated with centromeric chromatin and participate in the assembly of functional kinetochores (Henikoff 2001; McNulty and Sullivan 2018). Intriguingly, our findings reveal that PbaSat01-182 shares high sequence similarity with the 174 bp *Hae*III element, a centromeric satDNA (CENSTRIG) isolated from *K*. *blakistoni* (Yamada et al. 2004)*.* This centromeric satDNA was previously recognized as specific to the Strigidae family, as it is conserved across multiple Strigidae genera but completely absent in *T. alba* (Yamada et al. 2004). The discovery of a homolog satDNA in *P. badius* indicates that the 174 bp *Hae*III (CENSTRIG) element has a deeper evolutionary origin within Strigiformes than previously recognized. This shared ancestry, coupled with the apparent absence or complete turnover of this sequence in *Tyto* spp., suggests a heterogeneous centromeric repeat composition, a phenomenon often interpreted as evidence of the rapid evolutionary turnover of centromeric DNA (Plohl et al. 2008).

## Conclusion

This study presents the first karyotype description of *T. furcata* and expands current knowledge of genome organization and repetitive DNA evolution in Tytonidae through an integrated cytogenetic and repeatome-based approach. The strong karyotypic similarity between *T*. *furcata* and *T*. *alba*, including the conserved diploid number and chromosome morphology, indicates remarkable chromosomal stability within the genus despite millions of years of evolutionary divergence. At the same time, the elevated diploid numbers observed in Tytonidae relative to the ancestral avian condition support the hypothesis that extensive chromosomal fissions shaped the evolutionary history of this family.

Comparative analyses of repetitive DNA revealed substantial divergence in satellitome composition among the studied species, demonstrating that repetitive sequences can evolve rapidly even in the absence of major macrostructural chromosomal rearrangements. The identification of shared and species-specific satDNA families between *T*. *furcata* and *T*. *alba* is consistent with the library hypothesis, suggesting differential amplification and contraction of ancestral repeats following speciation. In *P*. *badius*, the strong association between satDNAs and transposable elements indicates that mobile elements may have played a significant role in satellitome diversification and genome evolution.

Overall, this work demonstrates that conserved karyotypic architecture can coexist with repetitive DNA diversification and emphasizes the importance of integrating cytogenetics and genomics to better understand chromosome evolution in birds. These findings provide an important framework for future comparative studies investigating genome evolution, centromere dynamics, and sex chromosome differentiation in Strigiformes.

## Supplementary Information

Below is the link to the electronic supplementary material.

Supplementary file1 (DOCX 655 KB)

## Data Availability

All data supporting the findings of this study are available within the paper and its Supplementary Information. The raw reads were deposited in the Sequence Read Archive (SRA-NCBI) under accessions numbers SRR38417748 (female individual) and SRR38417747 (male individual). All satDNA catalogs were deposited on the Genbank with accession numbers: PZ387877 to PZ387921.

## References

[CR1] Aliabadian M, Alaei-Kakhki N, Mirshamsi O, Nijman V, Roulin A (2016) Phylogeny, biogeography, and diversification of barn owls (Aves: Strigiformes). Biol J Linn Soc 119:904–918. 10.1111/bij.12824

[CR2] Altschul SF, Gish W, Miller W, Myers EW, Lipman DJ (1990) Basic local alignment search tool. J Mol Biol 215:403–410. 10.1016/S0022-2836(05)80360-2 2231712

[CR3] Belterman RHR, De Boer LEM (1984) A karyological study of 55 species of birds, including karyotypes of 39 species new to cytology. Genetica 65:39–82. 10.1007/bf00056765

[CR4] Bian X, Cai H, Ning S, Li Q, Zhang H, Xong X, Han L, Chen X, Liu A, Yang L (1991) Studies on the karyotypes of birds: XII. 15 species of nonpasserines (Aves). Zool Res 12:407–412

[CR5] Bolger AM, Lohse M, Usadel B (2014) Trimmomatic: a flexible trimmer for Illumina sequence data. Bioinformatics 30:2114–2120. 10.1093/bioinformatics/btu170 24695404 PMC4103590

[CR6] Cara LB, Levine MT (2021) Functional diversification of chromatin on rapid evolutionary timescales. Annu Rev Genet 55:401–425. 10.1146/annurev-genet-071719-020301 34813351 PMC9235829

[CR7] Cioffi MB, Toma GA, Setti PG, Vidal JAD, Mota-Souza G, Altmanová M, de Oliveira EHC, Kretschmer R (2026) Bridging methodological gaps in avian cytogenetics: comprehensive and optimized protocols for chromosomal preparation in birds. Chromosome Res 34:11. 10.1007/s10577-026-09803-8 42144513 PMC13180766

[CR8] Daugherty RL, Iii AJB, Salas VG, Contreras FJ, Chávez R, Mendes GC, Nestler JR, Johnson DH (2026) Morphological assessment of the Common Barn Owl *Tyto alba* (Strigiformes: Tytonidae) supports three species. Zootaxa 5752:399–416. 10.11646/zootaxa.5752.3.5 42408893

[CR9] De Coster W, D’Hert S, Schultz DT, Cruts M, Broeckhoven CV (2018) NanoPack: visualizing and processing long-read sequencing data. Bioinformatics 34:2666–2669. 10.1093/bioinformatics/bty149 29547981 PMC6061794

[CR10] Degrandi TM, Barcellos SA, Costa AL, Garnero ADV, Hass I, Gunski RJ (2020) Introducing the Bird Chromosome Database: an overview of cytogenetic studies in birds. Cytogenet Genome Res 160:199–205. 10.1159/000507768 32369809

[CR11] Ellegren H (2010) Evolutionary stasis: the stable chromosomes of birds. Trends Ecol Evol 25(5):283–291 20363047 10.1016/j.tree.2009.12.004

[CR12] Faria R, Navarro A (2010) Chromosomal speciation revisited: rearranging theory with pieces of evidence. Trends Ecol Evol 25:660–669. 10.1016/j.tree.2010.07.008 20817305

[CR13] Fry K, Salser W (1977) Nucleotide sequences of HS-alpha satellite DNA from kangaroo rat *Dipodomys ordii* and characterization of similar sequences in other rodents. Cell 12:1069–1084. 10.1016/0092-8674(77)90170-2 597857

[CR14] Garrido-Ramos MA (2017) Satellite DNA: an evolving topic. Genes (Basel) 8:230. 10.3390/genes8090230 28926993 PMC5615363

[CR15] Gill F, Donsker D, Rasmussen P (2025) IOC World Bird List (v15.1). https://www.worldbirdnames.org/new/. Accessed 25 March 2026

[CR16] Goubert C, Modolo L, Vieira C, Moro CV, Mavingui P, Boulesteix M (2015) De novo assembly and annotation of the Asian tiger mosquito (*Aedes albopictus*) repeatome with dnaPipeTE from raw genomic reads and comparative analysis with the yellow fever mosquito (*Aedes aegypti*). Genome Biol Evol 7:1192–1205. 10.1093/gbe/evv050 25767248 PMC4419797

[CR17] Griffin DK, Burt DW (2014) All chromosomes great and small: 10 years on. Chromosome Res 22:1–6. 10.1007/s10577-014-9413-0 24700106

[CR18] Griffin DK, Robertson LBW, Tempest HG, Skinner BM (2007) The evolution of the avian genome as revealed by comparative molecular cytogenetics. Cytogenet Genome Res 117:64–77. 10.1159/000103166 17675846

[CR19] Griffin DK, Kretschmer R, Larkin DM, Srikulnath K, Singchat W, Narushin VG, O’Connor RE, Romanov MN (2025) Avian cytogenomics: small chromosomes, long evolutionary history. Genes (Basel) 16:1001. 10.3390/genes16091001 41009947 PMC12470241

[CR20] Guerra MS (1986) Reviewing the chromosome nomenclature of Levan et al. Revista Brasileira de Genética 4:741–743

[CR21] Hassan HA (1998) Karyological studies on six species of birds. Cytologia 63:349–363. 10.1508/cytologia.63.349

[CR22] Henikoff S (2001) The centromere paradox: stable inheritance with rapidly evolving DNA. Science 293:1098–1102. 10.1126/science.1062939 11498581

[CR23] Kapusta A, Suh A (2017) Evolution of bird genomes-a transposon’s-eye view. Ann N Y Acad Sci 1389:164–185. 10.1111/nyas.13295 27997700

[CR24] Kimura M (1980) A simple method for estimating evolutionary rates of base substitutions through comparative studies of nucleotide sequences. J Mol Evol 16:111–120. 10.1007/BF01731581 7463489

[CR25] Kobayashi T (2011) Regulation of ribosomal RNA gene copy number and its role in modulating genome integrity and evolutionary adaptability in yeast. Cell Mol Life Sci 68:1395–1403. 10.1007/s00018-010-0613-2 21207101 PMC3064901

[CR26] König C, Weick F, Becking JH (2008) Owls of the world. Christopher Helm, London

[CR27] Kretschmer R, Ferguson-Smith MA, de Oliveira EHC (2018a) Karyotype evolution in birds: from conventional staining to chromosome painting. Genes 9:181. 10.3390/genes9040181 29584697 PMC5924523

[CR28] Kretschmer R, de Lima VLC, de Souza MS, Costa AL, O’Brien PCM, Ferguson-Smith MA, de Oliveira EHC, Gunski RJ, Garnero ADV (2018b) Multidirectional chromosome painting in *Synallaxis** frontalis* (Passeriformes, Furnariidae) reveals high chromosomal reorganization, involving fissions and inversions. Comp Cytogen 12:97–110. 10.3897/CompCytogen.v12i1.22344 PMC5904361 29675139

[CR29] Kretschmer R, Santos MS, Furo IO, Oliveira EHC, Cioffi MB (2022) FISH–in birds’. In: Liehr T (ed) Cytogenetics and molecular cytogenetics. CRC Press, Boca Raton, FL, USA, p 17

[CR30] Kumar S, Suleski M, Craig JE, Kasprowicz AE, Sanderford M, Li M, Stecher G, Hedges SB (2022) TimeTree 5: an expanded resource for species divergence times. Mol Biol Evol 39:msac174. 10.1093/molbev/msac174 35932227 PMC9400175

[CR31] Lawrence M, Huber W, Pagès H, Aboyoun P, Carlson M, Gentleman R, Morgan MT, Carey VJ (2013) Software for computing and annotating genomic ranges. PLoS Comput Biol 9(8):e1003118. 10.1371/journal.pcbi.1003118 23950696 PMC3738458

[CR32] Li H (2012) seqtk: toolkit for processing sequences in FASTA/Q formats. GitHub. https://github.com/lh3/seqtk

[CR33] Lui R, Blanco D, Moreira-Filho O, Margarido V (2012) Propidium iodide for making heterochromatin more evident in the C-banding technique. Biotechnic Histochem 87:433–438. 10.3109/10520295.2012.696700 22747174

[CR34] McNulty SM, Sullivan BA (2018) Alpha satellite DNA biology: finding function in the recesses of the genome. Chromosome Res 26:115–138. 10.1007/s10577-018-9582-3 29974361 PMC6121732

[CR35] Morgulis A, Gertz EM, Schäffer AA, Agarwala R (2006) A fast and symmetric DUST implementation to mask low-complexity DNA sequences. J Comput Biol 13(5):1028–1040. 10.1089/cmb.2006.13.1028 16796549

[CR36] Nishida-Umehara C, Tsuda Y, Ishijima J, Ando J, Fujiwara A, Matsuda Y, Griffin DK (2007) The molecular basis of chromosome orthologies and sex chromosomal differentiation in palaeognathous birds. Chromosome Res 15:721–734. 10.1007/s10577-007-1157-7 17605112

[CR37] Nurk S, Koren S, Rhie A et al (2022) The complete sequence of a human genome. Science 376:44–53. 10.1126/science.abj6987 35357919 PMC9186530

[CR38] O’Connor RE, Kiazim LG, Skinner BM, Fonseka G, Joseph S, Jennings R, Larkin DM, Griffin DK (2019) Patterns of microchromosome organization remain highly conserved throughout avian evolution. Chromosoma 128:21–29. 10.1007/s00412-018-0685-6 30448925 PMC6394684

[CR39] O’Connor RE, Kretschmer R, Romanov MN, Griffin DK (2024) A bird’s-eye view of chromosomic evolution in the class Aves. Cells 13:310. 10.3390/cells13040310 38391923 PMC10886771

[CR40] Peona V, Palacios-Gimenez OM, Blommaert J, Liu J, Haryoko T, Jønsson KA, Irestedt M, Zhou Q, Jern P, Suh A (2021) The avian W chromosome is a refugium for endogenous retroviruses with likely effects on female-biased mutational load and genetic incompatibilities. Phil Trans R Soc B 376:20200186. 10.1098/rstb.2020.0186 34304594 PMC8310711

[CR41] Plohl M, Luchetti A, Mestrović N, Mantovani B (2008) Satellite DNAs between selfishness and functionality: structure, genomics and evolution of tandem repeats in centromeric (hetero)chromatin. Gene 409:72–82. 10.1016/j.gene.2007.11.013 18182173

[CR42] Plohl M, Metrovic N, Mravinac B (2012) Satellite DNA evolution. Genome Dyn 7:126–152. 10.1159/000337122 22759817

[CR43] Poignet M, Johnson PM, Altmanová M, Majtánová Z, Dedukh D, Albrecht T, Reif J, Osiejuk TS, Reifová R (2021) Comparison of karyotypes in two hybridizing passerine species: conserved chromosomal structure but divergence in centromeric repeats. Front Genet 12:768987. 10.3389/fgene.2021.768987 34938317 PMC8687609

[CR44] Renzoni A, Vegni-Talluri M (1966) The karyograms of some Falconiformes and Strigiformes. Chromosoma 20:133–150. 10.1007/bf00335203

[CR45] Ribas MS, Azambuja M, Nogaroto V, Vicari MR (2026) Emergence of satellite DNAs suggests centromeric repositioning as a driver of karyotypic variation of the freshwater darter characines (*Apareiodon affinis*). Chromosome Res 34:2. 10.1007/s10577-026-09793-7 41718797 PMC12923391

[CR46] Rieseberg LH (2001) Chromosomal rearrangements and speciation. Trends Ecol Evol 16:351–358. 10.1016/s0169-5347(01)02187-5 11403867

[CR47] Ruiz-Ruano FJ, López-León MD, Cabrero J, Camacho JPM (2016) High-throughput analysis of the satellitome illuminates satellite DNA evolution. Sci Rep 6:28333. 10.1038/srep28333 27385065 PMC4935994

[CR48] Sambrook J, Russell DW (2001) Molecular cloning: a laboratory manual. Cold Spring Harbor Laboratory Press, New York, NY, USA

[CR49] Satović E, Vojvoda Zeljko T, Luchetti A, Mantovani B, Plohl M (2016) Adjacent sequences disclose potential for intra-genomic dispersal of satellite DNA repeats and suggest a complex network with transposable elements. BMC Genomics 17:997. 10.1186/s12864-016-3347-1 27919246 PMC5139131

[CR50] Schartl M, Schmid M, Nanda I (2016) Dynamics of vertebrate sex chromosome evolution: from equal size to giants and dwarfs. Chromosoma 125:553–571. 10.1007/s00412-015-0569-y 26715206

[CR51] Schmieder R, Edwards R (2011) Quality control and preprocessing of metagenomic datasets. Bioinformatics 27:863–864. 10.1093/bioinformatics/btr026 21278185 PMC3051327

[CR52] Setti PG, Deon GA, Dos Santos RZ, Goes CAG, Garnero ADV, Gunski RJ, de Oliveira EHC, Porto-Foresti F, de Freitas TRO, Silva FAO, Liehr T, Utsunomia R, Kretschmer R, Cioffi MB (2024) Evolution of bird sex chromosomes: a cytogenomic approach in Palaeognathae species. BMC Ecol Evol 24:51. 10.1186/s12862-024-02230-5 38654159 PMC11036779

[CR53] Sim SB, Corpuz RL, Simmonds TJ, Geib SM (2022) Hifiadapterfilt, a memory efficient read processing pipeline, prevents occurrence of adapter sequence in PacBio HiFi reads and their negative impacts on genome assembly. BMC Genomics 23:157. 10.1186/s12864-022-08375-1 35193521 PMC8864876

[CR54] Smit A, Hubley R, Green P (2020) RepeatMasker Open-4.0. http://www.repeatmasker.org. Accessed 20 Feb 2026

[CR55] Sproul JS, Hotaling S, Heckenhauer J, Powell A, Marshall D, Larracuente AM, Kelley JL, Pauls SU, Frandsen PB (2023) Analyses of 600+ insect genomes reveal repetitive element dynamics and highlight biodiversity-scale repeat annotation challenges. Genome Res 33:1708–1717. 10.1101/gr.277387.122 37739812 PMC10691545

[CR56] Sumner AT (1972) A simple technique for demonstrating centromeric heterochromatin. Exp Cell Res 75:304–306. 10.1016/0014-4827(72)90558-7 4117921

[CR57] Toma GA, dos Santos N, dos Santos R, Rab P, Kretschmer R, Ezaz T, Bertollo LAC, Liehr T, Porto-Foresti F, Hatanaka T, Tanomtong A, Utsunomia R, Cioffi MB (2023) Cytogenetics meets genomics: cytotaxonomy and genomic relationships among color variants of the Asian arowana *Scleropages formosus*. Int J Mol Sci 24(10):9005. 10.3390/ijms24109005 37240350 PMC10219274

[CR58] Ugarkovic D (2005) Functional elements residing within satellite DNAs. EMBO Rep 6(11):1035–1039. 10.1038/sj.embor.7400558 PMC1371040 16264428

[CR59] Uva V, Päckert M, Cibois A, Fumagalli L, Roulin A (2018) Comprehensive molecular phylogeny of barn owls and relatives (Family: Tytonidae), and their six major Pleistocene radiations. Mol Phylogenet Evol 125:127–137. 10.1016/j.ympev.2018.03.013 29535030

[CR60] Yamada K, Nishida-Umehara C, Matsuda Y (2004) A new family of satellite DNA sequences as a major component of centromeric heterochromatin in owls (Strigiformes). Chromosoma 112:277–287. 10.1007/s00412-003-0267-z 14997323

[CR61] Zattera ML, Bruschi DP (2022) Transposable elements as a source of novel repetitive DNA in the eukaryote genome. Cells 11:3373. 10.3390/cells11213373 36359770 PMC9659126

[CR62] Zhou Q, Zhang J, Bachtrog D, An N, Huang Q, Jarvis ED, Gilbert MTP, Zhang G (2014) Complex evolutionary trajectories of sex chromosomes across bird taxa. Science 346:1246338. 10.1126/science.1246338 25504727 PMC6445272

