# Supplementary material for: Satellite DNA evolution in Tytonidae (Aves: Strigiformes): dynamic repeat landscapes despite conserved karyotypes
Source: Chromosome Res. 2026 Sep 17;34(1):24. doi: 10.1007/s10577-026-09817-2 (PMC13582039; doi:10.1007/s10577-026-09817-2)
Supplement: Supplementary file 1 [file 10577_2026_9817_MOESM1_ESM.docx]

**Satellite DNA evolution in Tytonidae (Aves: Strigiformes): dynamic repeat landscapes despite conserved karyotypes**

Felipe Lagreca Bitencourt^1^, Gustavo Akira Toma^2^, Luciano Cesar Pozzobon^1^, Victor Cruz Cuervo^1^, Ricardo Utsunomia^3^, Maria Eduarda Gisloti-Ribeiro^3^, Michel Octávio Fiorenço Capana^3^, Raqueli Teresinha França^4^, Marcelo de Bello Cioffi^2^, Thales Renato Ochotorena de Freitas^1^, Rafael Kretschmer^1*^

^1^Laboratório de Citogenética e Evolução, Departamento de Genética, Instituto de Biociências, Universidade Federal do Rio Grande do Sul, Porto Alegre 91509-900, RS, Brazil

^2^Laboratório de Citogenética Evolutiva, Departamento de Genética e Evolução, Universidade Federal de São Carlos, São Carlos 13565-905, Brazil

^3^Faculdade de Ciências, Universidade Estadual Paulista, Bauru 13506-900, SP, Brazil

^4^Departamento de Clínicas Veterinária, Faculdade de Veterinária, Universidade Federal de Pelotas, Pelotas, RS, Brazil

* Correspondence: rafael.kretschmer@ufrgs.br

**Supplementary Information**

**Supplementary Table 1**. List of primers used for amplification satDNAs.

| **TfuSatsDNAs** | **Forward primer** | **Reverse Primer** |
| --- | --- | --- |
| TfuSat01-162 | AGCATGGTAGTTTGGAGGAA | TCTAGCTGCTTCCTTTCACT |
| TfuSat05-74 | ATAGCTTTGATTGCAGGATG | AAACTCCAAGTCTGGCGA |
| TfuSat06-80 | TCGTGGACCATTTTTGCG | TTGTCCCAAATCTATCCCAT |
| TfuSat07-66 | CTTGTCCTCCCGTAAGCG | CAGGGACGAACAGGTCTA |
| TfuSat08-267 | GGGAAGATTTGACAACGTAA | AATAGAGAGAGGCTTTTGTC |

**Supplementary Table 2.** Termocycle program used to amplify the satDNA primers from *Tyto furcata* (TfuSat).

| **SatDNA** | **Desnaturation** | **Cycles** | **Desnaturation** | **Annealing** | **Amplification** | **Final amp.** | **Concentration (ng/ul)** |
| --- | --- | --- | --- | --- | --- | --- | --- |
| TfuSat01-162 | 95 ºC (5 min) | 40 | 95 ºC (40 s) | 55 ºC (35 s) | 72 ºC (1 min) | 72 ºC (7 min) | 0,1 |
| TfuSat02-28 | Biotinylated Oligonucleotide | | | | | | |
| TfuSat03-28 | Biotinylated Oligonucleotide | | | | | | |
| TfuSat04-21 | Biotinylated Oligonucleotide | | | | | | |
| TfuSat05-74 | 95 ºC (5 min) | 40 | 95 ºC (40 s) | 54 ºC (35 s) | 72 ºC (1 min) | 72 ºC (7 min) | 0,1 |
| TfuSat06-80 | Not amplified | | | | | | |
| TfuSat07-66 | 95 ºC (5 min) | 40 | 95 ºC (40 s) | 55 ºC (35 s) | 72 ºC (1 min) | 72 ºC (7 min) | 0,1 |
| TfuSat08-267 | Not Amplified | | | | | | |

**Supplementary Table 3.** Characterization of satDNA families of *Tyto furcata*, showing the repeat unit length (RUL), the adenine/thymine content (A+T %), the divergence, the abundance in the female (F) and male (M) genomes, and the abundance of female/male proportion (F/M). SF=Superfamily.

| **SatDNA family** | **RUL (bp)** | **A+T (%)** | **Abundance, % (F)** | **Abundance, % (M)** | **Abundance (F/M)** | **Divergence (%)** | **SF** |
| --- | --- | --- | --- | --- | --- | --- | --- |
| TfuSat01-162 | 162 | 50.6 | 2.3263 | 2.4796 | 0.94 | 17.87 | - |
| TfuSat02-28 | 28 | 55.6 | 1.4864 | 1.4469 | 1.03 | 6.56 | - |
| TfuSat03-28 | 28 | 39.3 | 0.0801 | 0.1303 | 0.62 | 12.02 | - |
| TfuSat04-21 | 21 | 23.8 | 0.0440 | 0.0137 | 3.21 | 5.91 | SF-5 |
| TfuSat05-74 | 74 | 45.9 | 0.0378 | 0.0054 | 6.9 | 9.97 | - |
| TfuSat06-80 | 80 | 58.7 | 0.0128 | 0.0039 | 3.24 | 11.62 | - |
| TfuSat07-66 | 66 | 36.4 | 0.0066 | 0.0027 | 2.55 | 10.33 | - |
| TfuSat08-267 | 267 | 64.8 | 0.0025 | 0.0035 | 0.72 | 9.55 | - |
| **Total** | - | - | 3.9969 | 4.0862 | - | - | - |

**Supplementary Table 4.** Characterization of satDNA families of *Tyto alba*, showing the repeat unit length (RUL), the adenine/thymine content (A+T %), the divergence, the abundance in the female (F) and male (M) genomes, and the abundance of female/male proportion (F/M). SF=Superfamily.

| **SatDNA family** | **RUL (bp)** | **A+T (%)** | **Abundance, % (F)** | **Abundance, % (M)** | **Abundance (F/M)** | **Divergence (%)** | **SF** |
| --- | --- | --- | --- | --- | --- | --- | --- |
| TalSat1-42 | 42 | 28.6 | 2.5525 | 2.5564 | 1.00 | 12.75 | SF-5 |
| TalSat2-162 | 162 | 50.6 | 1.4116 | 0.9458 | 1.49 | 6.47 | - |
| TalSat3-21 | 21 | 24 | 1.0926 | 1.1264 | 0.97 | 14.73 | SF-5 |
| TalSat4-28 | 28 | 39.2 | 0.4791 | 0.4528 | 1.06 | 8.52 | - |
| TalSat5-28 | 28 | 54 | 0.4791 | 0.4900 | 0.98 | 8.51 | - |
| TalSat6-66 | 66 | 36.3 | 0.1299 | 0.0811 | 1.60 | 9.53 | - |
| TalSat7-1316 | 1316 | 30 | 0.0206 | 0.0250 | 0.82 | 7.11 | - |
| TalSat8-145 | 145 | 54.4 | 0.0110 | 0.0120 | 0.91 | 14.25 | SF-4 |
| TalSat9-262 | 262 | 23 | 0.0106 | 0.0126 | 0.85 | 3.58 | - |
| **Total** | - | - | 6.1874969 | 5.7023 | - | - | - |

**Supplementary Table 5.** Characterization of satDNA families of *Phodilus badius*, showing the repeat unit length (RUL), the adenine/thymine content (A+T %), the divergence and the relative abundance in the genome. SF=Superfamily.

| **SatDNA family** | **RUL (bp)** | **A+T (%)** | **Abundance, %** | **Divergence (%)** | **SF** |
| --- | --- | --- | --- | --- | --- |
| PbaSat01-182 | 182 | 45.6% | 1.8726 | 2.39 | - |
| PbaSat02-1308 | 1308 | 44.8% | 1.2205 | 14.62 | - |
| PbaSat03-729 | 729 | 55.8% | 0.3876 | 1.91 | SF-1 |
| PbaSat04-241 | 241 | 74.7% | 0.2472 | 8.48 | - |
| PbaSat05-36 | 36 | 30.6% | 0.2324 | 10.15 | - |
| PbaSat06-1115 | 1115 | 47.9% | 0.1731 | 3.49 | - |
| PbaSat07-52 | 52 | 40.4% | 0.1106 | 13.04 | SF-2 |
| PbaSat08-53 | 53 | 35.8% | 0.0920 | 12.92 | SF-2 |
| PbaSat09-146 | 146 | 45.9% | 0.0823 | 8.62 | SF-4 |
| PbaSat10-231 | 231 | 58.9% | 0.0562 | 11.89 | SF-1 |
| PbaSat11-1256 | 1256 | 53.6% | 0.0502 | 3.61 | SF-1 |
| PbaSat12-193 | 193 | 46.1% | 0.0489 | 8.68 | SF-3 |
| PbaSat13-553 | 553 | 57.3% | 0.0436 | 6.95 | SF-1 |
| PbaSat14-190 | 190 | 45.3% | 0.0422 | 10.21 | - |
| PbaSat15-544 | 544 | 55.7% | 0.0384 | 7.48 | SF-1 |
| PbaSat16-207 | 207 | 56.5% | 0.0332 | 10.38 | SF-1 |
| PbaSat17-24 | 24 | 41.7% | 0.0310 | 8.14 | - |
| PbaSat18-38 | 38 | 47.4% | 0.0202 | 3.78 | - |
| PbaSat19-47 | 47 | 29.8% | 0.0181 | 10.85 | - |
| PbaSat20-1011 | 1011 | 52.2% | 0.0171 | 4.60 | SF-1 |
| PbaSat21-35 | 35 | 45.7% | 0.0170 | 21.76 | - |
| PbaSat22-254 | 254 | 75.2% | 0.0156 | 8.89 | - |
| PbaSat23-190 | 190 | 45.3% | 0.0147 | 5.38 | SF-3 |
| PbaSat24-27 | 27 | 63.0% | 0.0096 | 8.21 | - |
| PbaSat25-176 | 176 | 53.4% | 0.0086 | 10.26 | - |
| PbaSat26-44 | 44 | 40.9% | 0.0070 | 5.06 | - |
| PbaSat27-174 | 174 | 60.3% | 0.0063 | 12.11 | - |
| PbaSat28-30 | 30 | 40% | 0.0063 | 8.53 | - |
| **Total** | - | - | 4.8173789 | - | - |

**Supplementary Table 6**. Similarity between satDNA families from *T*. *alba* and *T. furcata*. Each row corresponds to a satDNA family, with orthologous sequences shown side by side. The similarity column indicates the percentage of identity between the two sequences. Superfamilies are represented by SF.

| ***Tyto alba*** | ***Tyto furcata*** | **Similarity (%)** | **SF** |
| --- | --- | --- | --- |
| TalSat03-21 | TfuSat04-21 | 100 | SF-5 |
| TalSat02-162 | TfuSat01-162 | 100 | - |
| TalSat04-28 | TfuSat03-28 | 100 | - |
| TalSat05-28 | TfuSat02-28 | 100 | - |
| TalSat06-66 | TfuSat07-66 | 100 | - |

**Supplementary Table 7.** BLAST-search results from *barn owls* satellitome against GenBank-NCBI database.

| **SatDNA family** | **BLAST-Sequence name** | **BLAST- Species ID** | **Query-cover (%)** | **E-value** | **Percentage identity (%)** | **Accession number** |
| --- | --- | --- | --- | --- | --- | --- |
| PbaSat03-729 | W chromosome repeat sequence | *Asio otus* | 100 | 1e-159 | 91.5 | AF231331.1 |
|  | W chromosome repeat sequence | *Bubo scandiacus* | 100 | 5e-158 | 91.2 | AF231326.1 |
|  | W chromosome repeat sequence | *Glaucidium passerinum* | 53 | 5e-143 | 91.2 | AF291661.1 |
| PbaSat12-193 | Tandem repeat P1 | *Ara ararauna* | 88 | 4e-64 | 94.1 | S48542.1 |
|  | Tandem repeat P1 | *Psittacara erythrogenys* | 88 | 6e-52 | 90 | S48543.1 |
|  | Tandem repeat P1 | *Amazona aestiva* | 46 | 4e-14 | 85.2 | S49011.1 |
| PbaSat14-190 | RBMII DNA with inverted repeat | *Anas platyrhinchos* | 100 | 4e-74 | 94.2 | AB604922.1 |
|  | RBMII DNA with inverted repeat | *Aix sponsa* | 84 | 1e-58 | 93.7 | X61410.1 |
|  | Centromeric repeat | *Anas platyrhinchos* | 96 | 1e-49 | 87.4 | LC416792.1 |
|  | Microssatelite MM01 | *Mergus merganser* | 90 | 5e-48 | 88.3 | AY679118.1 |
|  | RBMII DNA with inverted repeat | *Sarkidiornis melanotos* | 93 | 6e-47 | 87. | X61407.1 |
| PbaSat22-254 | Predicted-CCR9 transcript variant | *Strix aluco* | 89 | 1e-55 | 85 | XR_012625205.1 |
| PbaSat26-44 | Predicted-ncRNA | *Athene cunicularia* | 82 | 2e-06 | 97.2 | XR_003398700.1 |
| TalSat02-162 | Predicted-mRNA | *Tyto alba* | 94 | 1e-38 | 87 | XM_042799938.1 |
| TfuSat02-162 | Predicted-mRNA | *Tyto alba* | 94 | 1e-38 | 87 | XM_042799938.1 |

**Supplementary Table 8.** BLAST-search results from barn owls satellitome against RepBase/CENSOR database. Begin and end indicate the location of the repeat on the query sequence. Matching repeat indicates the identified motifs. Class/family represents the repeat’s major classification. Dir indicates orientation - Direct (d) or complementary (c). Sim = similarity score (decimal) and SW, contains blast alignment score.

| **satDNA**  **family** | **Begin (bp)** | **End (bp)** | **Matching repeat** | **Repeat class/family** | **Dir** | **Sim** | **SW score** |
| --- | --- | --- | --- | --- | --- | --- | --- |
| PbaSat-01-182 | 3 | 182 | CENSTRIG | Tandem repeat | d | 0.86 | 328 |
| PbaSat02-1308 | 26 | 1306 | CR1-J2_Pass | Interspersed repeat | d | 0.87 | 2670 |
|  |  |  |  |  |  |  |  |
|  |  |  |  |  |  |  |  |
|  |  |  |  |  |  |  |  |
|  |  |  |  |  |  |  |  |
|  |  |  |  |  |  |  |  |

Supplementary Table 9. Comparison of TalSatDNA genomic abundance estimated from Illumina short reads (male), PacBio HiFi long reads (mean ± SD of three 1.5 Gb subsamples, seeds 100/200/300), and the scaffold-level PacBio genome assembly (GCF_018691265.1), all from the same male individual.

| **SatDNA family** | **Illumina (male, %)** | **PacBio HiFi reads (mean ± SD, %)** | **Genome assembly (%)** | **Fold PacBio/Illumina** | **Fold Assembly/PacBio** |
| --- | --- | --- | --- | --- | --- |
| *TalSat1-42* | 2.5565 | 7.1000 ± 0.0592 | 0.0039 | 2.78× | 0.0005× |
| *TalSat2-162* | 0.9458 | 0.2421 ± 0.0180 | 0.0069 | 0.26× | 0.0285× |
| *TalSat3-21* | 1.1264 | 5.8694 ± 0.0424 | 0.0008 | 5.21× | 0.0001× |
| *TalSat4-28* | 0.4528 | 0.0011 ± 0.0008 | 0.0003 | 0.002× | 0.2647× |
| *TalSat5-28* | 0.4900 | 0.0004 ± 0.0002 | 0.0002 | 0.001× | 0.5455× |
| *TalSat6-66* | 0.0812 | 0.2240 ± 0.0165 | 0.0060 | 2.76× | 0.0268× |
| *TalSat7-1316* | 0.0250 | 0.0263 ± 0.0032 | 0.0048 | 1.05× | 0.1825× |
| *TalSat8-145* | 0.0121 | 0.0549 ± 0.0185 | 0.0019 | 4.55× | 0.0346× |
| *TalSat9-262* | 0.0126 | 0.0147 ± 0.0019 | 0.0012 | 1.16× | 0.0818× |
| **Total** | **5.7024** | **13.5329** | **0.0260** | **2.37×** | **0.0019×** |

**Supplementary Figure 1**. Repeat landscape from a female of *Tyto furcata* with satDNAs grouped in order of abundance.

**Supplementary Figure 2**. Repeat landscape from a female of *Tyto alba* with satDNAs grouped in order of abundance.

**Supplementary Figure 3**. Landscape of *Phodilus badius* with satDNAs grouped in order of abundance.
